# Career and Self-Construction of Emerging Adults: The Value of Life Designing

**DOI:** 10.3389/fpsyg.2015.02041

**Published:** 2016-01-11

**Authors:** Jacobus G. Maree, Adeline Twigge

**Affiliations:** Department of Educational Psychology, Faculty of Education, University of PretoriaPretoria, South Africa

**Keywords:** life design counseling, emerging adult, self-construction, narrative identity, spiritual development, interpersonal adaptability, future consciousness, citizen-leadership

## Abstract

This article describes a potential way of counseling emerging adults from a life design perspective to construct a self that could enable them to be agents of both their own development and the development of others. Theoretical issues relating to a dynamic, developmental and systems framework of the understanding of wellbeing are described and the process involved is delineated. The research design was qualitative and comprised case studies. Six participants who subscribed to the definition of “emerging adults” and were comparatively representative of the ethnic diversity of South Africa, were selected purposively from a group of individuals who applied for career counseling in a private practice context. The intervention involved life design counseling and occurred over a period of 6 weeks. Information related to participants' self-construction was gathered using qualitative techniques, including the *Career Interest Profile*, the Career Construction Interview, a timeline, video clips, a collage, and semi-structured interviews. Following the intervention, the participants revealed heightened insights with regard to aspects of their sense of a relational-moral self. Results indicated that life design counseling could enhance elaborative personal development (enhancing self-awareness and reaping the benefits of developing an improved relational-moral self) and the promotion of an awareness of the importance to promote social justice in work-related contexts.

## Introduction

Much has been written about the ripple effect of multiple changes in the world at large (and especially in the workplace), as well as the tremendous impact of change on humankind in general and on workers in particular (Guichard, 2013). Work environments are becoming unstable, while lifelong employment in one organization and regular promotion in that organization are no longer guaranteed. Therefore, employees no longer feel compelled to remain loyal to any one organization throughout their working lives (Maree and Di Fabio, 2015). While Krumboltz and Chan (2005), contend that it is crucially important to embrace change, Blustein (2006, 2011) has made repeated requests for a wide-ranging psychology of working. It is important for career counselors in particular to prepare their clients to not only *accept*, but in fact *welcome* change: “Expecting people to decide on a lifetime career and commit to that choice is a formula for personal disaster. Circumstances change, economic cycles have their booms and busts, technology advances, and people's interests change over time. Remaining ever open-minded is the smartest way to adapt to change” (Krumboltz and Chan, 2005, p. 351). The field of career counseling has no choice but to stay abreast of changes if it is to remain relevant (Blustein, 2011). Assessment and intervention should therefore be aimed at improving people's employability, enhancing their career adaptability, and helping them assume authorship of their career and life stories.

This view is consistent with the opinion expressed by Bernaud (2014, p. 36), namely that “career choices faced by individuals inevitably raise the question of the meaning that they intend to give their lives. To choose their work or sector in which they want to evolve, is also to consider the purpose of their existence, the priorities (physical, spiritual, social, aesthetic, etc.) that they want to give, the choices that they wish to operate, the overall style of life that they wish to give themselves” (Bernaud, 2014, p. 36). Clearly, career-related transitioning requires an awareness of the self so as to show a greater openness to the deeper dimensions of humaneness (Maslow and Lowry, 1968). Lombardo (2007) explains that although the concept of “wisdom” is often associated with knowledge constructed in the past—to be more specific, from wise men and philosophers—these “wise” people use such accumulated knowledge to deal with current and perceived future challenges and transitions related to career life. Career counseling, seen from this perspective, requires career counselors to equip their clients to act wisely. Wisdom, seen thus, strongly relates to a stronger future-oriented awareness and requires a profound understanding of behavioral tendencies and patterns. Lombardo (2007) regards wisdom as the highest expression of self-development, the synthesis and application of the virtues of an enhanced future awareness. It is the perpetually developing understanding of and fascination with both the bigger picture and the personal dimensions of life, of what is important, ethical and meaningful, and it involves the desire and creative ability to apply this understanding to design a better life (which includes finding a decent job) so as to benefit the self and others.

### Rationale for the study

While the need to help people become more career resilient and employable is a common topic in the educational research literature, very little has been written about the role of clients' spirituality in career-life counseling. In this article, the emphasis is therefore on the cardinal (yet largely neglected) importance of permanent spiritual values and ideals that clients should steer personally so as to enable them to construct their own identity, find decent work and design successful lives.

## Introducing a moratorium phase for emerging adults

Major work-related changes, as well as social and cultural changes over the past number of decades have spawned a postponement or “moratorium” phase among young people in the more well-off communities between the ages of 18 (when most of them complete their school careers) and 30 (before they enter the adult phase). They take longer (compared to the young persons of a few decades ago) to find their feet as far as stable accommodation, work and intimate relationships in particular are concerned (Harter, 2012; Facio and Resett, 2013). This period (18–30 years old) is considered the period of budding or emerging maturity, in imitation of authors such as Arnett (2012) and Smith et al. (2011).

An example of an initiative that correlates with the above scenario is the Western Cape Development Strategy (2013) that was launched by the Western Cape Department of Social Development (South Africa) in September 2013. The Western Cape Provincial Government recognized that local youths (14- to 25-year-olds) were part of a “youth bulge” that constituted the vast majority of the population in the province. Moreover, it realized that this group could be regarded either as a demographic asset or as a demographic time bomb, and therefore projected that within the next decade these youths would be either an asset or a threat to social stability, depending on the extent to which their current restrictive life opportunities would be optimalised via the development processes they go through. Plans were purposefully made and are being carried out to ensure that these youths take their rightful place in society instead of simply swelling the ranks of the unemployed. In terms of this strategy, the development of youth leaders is core to the handling of long-term challenges. Youth leadership, as understood in terms of social development, does not deal with narrow-minded “role model” definitions of leadership, but rather with the improvement and increase of a variety of services, support structures and different opportunities that are available to youths as leaders of change in their communities—initially as youths and later as adults. Moreover, the development of youth leaders is regarded as essential for youth participation in their own development processes, their communities and society at large. It is assumed that as the leadership capabilities and powers of youths are developed, this will over time have a fundamental impact on the social institutions with which they interact. This will cumulatively lead to the transformation and change of the negative local landscapes and settings that contribute so much to the different challenges currently faced by young people.

The designers of the Western Cape Development Strategy (2013) declare that well-grounded theoretical frameworks offer a good springboard and conclude that “the needs for evidence-based means to address the challenges of the twenty-first century will coalesce to make Lewin's (1951, p. 169) quote that ‘There is nothing so practical as a good theory’ an oft-proven empirical reality.” They argue that the science of development as a means of promoting social justice may well become the most significant lens through which to consider the future contributions of such science. They also contend that counseling should promote a moral orientation in the emerging adult in such a way that good will be created by way of contributions to positive person-context relationships. When emerging adults see themselves as people who are morally bound to and involved in the establishment of civil society, and when they consequently start to gain a transcendent sense of the importance of life as a commitment to something of an enduring nature or lying beyond the constraints of their own existence, they are empowered to be agents of both their own development and of the positive promotion of other persons and elements of society. A commitment with regard to spiritual action in a world outside of the self will encourage these young persons to bequeath to future generations a society characterized by social equality and democracy, as well as a world in which youths may flourish and stand a fair chance of finding a decent job.

Considering that the context of emerging adults is ever-changing, ongoing, and characterized by an interplay of forces, the complex context of emerging adults makes self-construction (Guichard, 2005, 2009) an appropriate avenue or lens by means of which to view, investigate, and interpret their idiosyncratic contextual situatedness. In cohesion with the above, it seems that career construction theory (Savickas, 2011) supports individuals with a new perspective on their life-career constructions, which prepares them to make wise choices and become involved in specific activities. More specifically, life design counseling promotes the deliberate and intentional preparation of emerging adults for an emotionally strong and healthy and a socially secure, satisfactory, and productive life (Maree, 2013).

Self-construction and life design counseling will now be discussed briefly.

### Self-construction theory

Self-construction theory regards people as proactive agents whose main activity is self- organization to maintain stability and continuousness in their career lives instead of being passive objects, subjected to the whims of external forces (at the mercy of fate; Mahoney, 2002). On a related note, Guichard and Lenz (2005) emphasize the importance of individuals' contexts by explaining that people construct themselves in a specific manner, which depends on the way in which they relate to themselves as entities subsisting at particular moments in time in specific societies. As multidimensional human beings in a postmodern society, people join their different career-life domains at any given time and structure them according to various projections (Guichard and Dauwalder, 2010). In life design counseling, the focus in terms of context is on the relationship between the individual and his/her environment, as well as on the interaction that occurs within this system with its subsystems (Zunker, 1998).

### Life design counseling

For the purposes of the current article, we focus on the life design framework and approach recently developed by Savickas et al. (2009).

#### The life design counseling framework of Savickas et al. (2009)

The use of narratives lies at the heart of life design counseling. Clients' narratives are utilized by the career counselor to understand their career-life experiences and events in life contexts (Maree, 2013). Clients use their own language (obviously shaped by life contexts) to construct narratives. Maree (2013) states that career counselors act as co-constructors of clients' life stories and facilitate the narration of their career-life stories by drawing attention to poignant themes and tensions in their career-life storylines. Furthermore, they teach them the skills they need to write and perform the next episode in their career-life story. By doing this, the career counselor augments clients' vocabulary, effectively equips them with the language they need to express themselves and elucidates their interpretation of their own environments. Savickas (2013) maintains that individuals' experiences in their families help them to devise and shape their social roles as actors. These roles are modified continuously as the need arises and as clients “act” out such roles on various career-life stages or theaters such as the family, school, community/society, tertiary training institution, and work-life contexts. During this process, clients in reality write and advance their autobiographical stories, which provide them with autobiographical bridges to deal with multiple transitions. Clients' career-life stories subsequently shed light on and facilitate stability and constancy in their lived experiences in work-life and related environments.

Life design counseling blends self- and career- construction (Savickas et al., 2009). Based on social constructionism, it recognizes that social interaction produces awareness of the self and an understanding of one's situatedness, while it also promotes identity formation. Moreover, conversation facilitates the co-construction of meaning in the lives of people. Life design counseling equips career counselors with the *savoir faire* to help their clients deal with the challenges and needs that they encounter and experience in their unique contexts. Significantly, the life design process not only focuses on the importance of deciding which career to choose; it also supports clients in devising adaptable career lives that can be reshaped as and when life experiences ensue and changes occur (Campbell and Ungar, 2004).

The ultimate aim of life design counseling is to bring about change in clients' career lives in respect of their adaptability, narratability, intentionality and, ultimately, action orientation. Whereas adaptability speaks of one's ability to deal with change, narratability alludes to continuousness in one's career-life story. Taken together, adaptability and narratabilty ensure suppleness and constancy of the sense of self and indicate an ability to participate in meaningful activities, to flourish in the twenty-first century knowledge society (despite having to make transitions on multiple occasions) and, ultimately, to move forward by dealing with existent activities. In doing so, clients uncover abilities and interests they would prefer to execute (Savickas et al., 2009). The value of life design counseling as such is implied in a counseling process that fulfills two functions at the same time, namely a reflection function and a design function.

Life design counseling promotes heuristic development by harnessing and merging client-centered, psychodynamic, and (aspects of) cognitive-behavioral approaches (Hill, 2009) to facilitate self-reflection and reflexivity (Guichard et al., 2011) and, ultimately, advance people's career-lives. The aim of exploring and building on the magic in the life stories of individuals is to compile a life portrait that does not only transform the individual's life, but also makes a difference in the broader context. Such a “relational-moral self” is probably described best in Buechner's (1993, p. 119) distinctive statement and the observation made by Savickas (2006, 2011, p. 33). Buechner considers man's life journey as a response to a calling and states: “The place God calls you to is the place where your deep gladness and the world's deep hunger meet,” while Savickas remarks, in support, that something special (magic) occurs when there is movement: “Magic happens when we move [forward].”

It seems that life design counseling may contribute to a situation in which emerging adults can play a role in creating a favorable environment in which young persons may flourish. Exactly how this initiation should take place is uncertain and this was indicated as a shortcoming in the literature. The working assumption is that life design counseling (with the narrative as the vehicle that helps to extend the autonomous self to a “self-in-context” that displays a “self-others” orientation—in other words that contributes to the greater good) lends itself to bridging this gap.

### Goals of the study

Our goal was to explore the value that life design counseling holds for the career construction and self-construction of emerging adults. Our specific research questions were:

- How is life design counseling experience by emerging adults who seek career counseling?
- How can life design counseling enhance the personal development of emerging adults (enhancing self-awareness and reaping the benefits of developing an improved relational-moral self) in a way that enhances career and self-construction?

## Methods

### Research design

The research design was of a qualitative nature and involved an intrinsic, instrumental, collective case study. An interpretivist paradigm was utilized, which expedited a thorough understanding and profound interpretation of meanings revealed during our interactions with clients. An explorative, descriptive, collective case study was implemented to facilitate an in-depth investigation of the outcomes of life design counseling with regard to the self-construction of emerging adults (McMillan and Schumacher, 2009). The reason for our choice was that we believed it would enable us to study the constructs of life design counseling and career adaptability from multiple perspectives within the context in which it occurred (Creswell, 2013).

### Case description

Selection criteria called for participants in the “new life phase” group (the so-called emerging adults) so that we would be able to understand the relational-moral relationships of emerging adults by obtaining a holistic picture of the interaction between the subsystems (life roles and different life contexts) at play during self-construction. Multidimensional data regarding the emerging adult as a relational-moral career constructor and community citizen or community leader was gathered and analyzed.

Given that it was our aim to examine the career-related challenges faced by people with some degree of choice in their lives, the following selection criteria applied. First, participants had to be between the ages of 18 and 30. Second, they had to come from the middle- and higher-income environment (and therefore had some degree of choice in their lives). Third, participants must have displayed a need for career counseling. Fourth, participants had to be literate and verbally developed enough to make a constructive contribution to the research project (academic performance and an interview were used to determine whether the candidate was suitable or not). Fifth, an equal number of participants who speak Afrikaans, English, and an African language had to be involved. Lastly, participants had to agree to participate in the life design counseling intervention. The objective was to investigate the value of life design counseling in the construction of a relational-moral self for each of these six emerging adults. Table 1 contains a summative overview of the background information regarding the participants.

**Table 1:** **A summative overview of the background information regarding the participants**.

**PARTICIPANT**	**1**	**2**	**3**	**4**	**5**	**6**
Age	22	28	20	19	30	29
Gender	Female	Male	Male	Female	Female	Male
Ethnicity	White	White	Colored	Colored	Black	Black
Mother tongue	Afrikaans	Afrikaans	English	English	Southern Sotho	Yoruba
Highest qualification	BA with French and German	Matric (needs one subject for degree in architecture)	Matric	Matric	BTech Auditing	National Diploma in Banking and Management
Student, employee, entrepreneur or unemployed	Student	Employee and entrepreneur	Employee	Unemployed	Unemployed	Employee and entrepreneur
Accommodation arrangements	Independent from parent	Lives with parents	Lives with parents	Lives with parent	Lives with parent	Independent

Despite the fact that the participants apparently wanted help “only” with their choice of a suitable occupation, they frequently put forward broader issues that they wanted to sort out. These broader issues implied an approach that necessitated the design of a life career and that further highlighted an investigation into the possible value of life design counseling.

Participant 1 had already completed a degree, but she also wanted to specialize in a medical field (area still to be discerned)—something that would require many more years of training. She wished to reconcile her vision of a satisfactory and fulfilling life career with a bigger picture that could reveal a potential that had previously been obscured to her. Participant 2 wanted to eliminate potential traps from his thought processes since he considered sacrificing the security of an existing job opportunity in order to strengthen his position as entrepreneur. Participant 3 requested help with his career choice, but at the same time wished to gain greater self-knowledge that would support him with his plans for the future. Participant 4's need for life career counseling involved the choice of a suitable occupation, since it was a year since she matriculated and she had still not managed to take a clear course. Despite having already been well-qualified, Participant 5 experienced a search for herself, which she defined in more detail as a lack of specific sense and meaning (being productive, constructive and creative). Participant 6 probably experienced a lack of focus, since he not only had numerous interests, but also was particularly eager to learn. Hence, it transpired that he wished to align his passion or vocation with a suitable occupation in order to conclude the entire process in a focused manner.

Various strategies were used to ensure crystallization, while trustworthiness was ensured through the use of triangulation during the data collection and analysis phases (McMillan and Schumacher, 2009; Creswell, 2013).

#### Credibility

To ensure credibility of the data and appraise contradictory data, we used triangulation, and crystallization (we focused on the emergence of multifaceted patterns and themes). Moreover, we ensured peer review by collaborating with a colleague who was indifferent and impartial to the study. She (our external coder) coded all data independently to make sure that the identified themes were a true representation of the data. In addition, participant review was facilitated in that we made sure that the participants had the opportunity to decide whether the results and inferences were correct. We cleared up any possible misunderstandings and concepts discussed with the participants before interpreting and analysing the data.

#### Transferability

We based our research on comprehensive descriptions of the case study and made no attempt to generalize. We ensured that any inferences were supported by sufficient evidence from the data. We documented detailed descriptions of the research setting and the techniques used to ensure that sufficient information existed on the context of events.

#### Confirmability

We avoided selective use of data and fully documented the methods used and the decisions made during the study.

#### Dependability

The data was reported verbatim. Information was presented in as much detail as possible (including information potentially contradictory to the identified themes, subthemes, and subsubthemes). We maintained an audit trail by carefully recording all communications, and we communicated with participants in their language of communication.

#### Triangulation and crystallization

Richardson (1997) advocates the use of the term crystallization rather than triangulation in qualitative research. Different qualitative data-gathering methods were therefore used to facilitate crystallization and enhance the trustworthiness of the study.

### Intervention

The intervention (individual narrative career counseling) comprised six life design sessions. Each session comprised 45–60 min each over a period of 6 weeks.

The intervention is described in Table 2.

**Table 2:** **Description of the intervention**.

**Sessions**	**Life design intervention**	**Proposed content of sessions**	**Expected outcomes**
1	The problem was defined and the results that the participant hoped to achieve, were identified.	Maree's (2010) *Career Interest Profile* (*CIP*) with its 19 occupational categories was handed out and explained, after which the questionnaire was completed together with the participant. The completed questionnaire was subsequently used as the basis for a conversation about provisionally favored occupations, while at the same time the issue of meeting the ongoing needs of the greater good was touched upon.	Firstly, by creating an “appealing” environment, participants were motivated to participate in the life design intervention programme. Furthermore, the purpose of the activity was the identification and exploration of participants' interests, as well as the role of benevolence/goodwill (based on their opinion and understanding of their own life experiences). It was hoped that the participants would gain both self- and occupational knowledge through their deeper insight into matters that could be meaningful to themselves as well as in the broader contex.
2	The participant and researcher together investigated the participant's current scenario.	The career interview (the five primary domain questions) was used to start with the co-construction of an occupation (in the form of a new life story). During this process, the self-concept was linked to a preferred occupational environment, and at the same time core life problems and fixations dating from childhood were sorted out. A life slogan was employed to serve as leverage for launching a new chapter in the participant's life story. The ethical aspects were explored co-constructively with the help of life-counseling techniques.	
3	New scenarios were revealed as the researcher told the participant's story from a new perspective and revised it together with him or her.	The researcher expanded the participant's moral horizon by way of a timeline (covering the evolution of human consciousness—from caveman to postmodern mindfulness) and video inserts. While the first four (brief) video clips highlighted current and future socio-cultural and ecological crises, the next four dealt with the need to extend the self to others, the need for leaders who would be willing to become involved in finding solutions to socio-cultural and ecological crises as well as the value of cultivating and practicing wisdom. The focus was on possible contributions to meet current needs and serve the greater good, as well as on solutions for future society. The researcher made a connection between the meanings drawn from the participant's career story interview as well as further assessments and the initial reason for counseling: The initial career story was converted into a clear character sketch with an occupational theme.	Participants were given the opportunity to provide sense and meaning to their own lives. By taking into account the past and present (of their own lives as well as that of humankind), they were able to identify life themes that could be intertwined with future career-life ideals and that involved addressing the needs of the greater good, as well as providing solutions for the dilemmas of society.
4	The problem was placed within a new story.	Based on the information obtained from previous sessions, a collage was used to create—jointly and within the framework of a macro-narrative or life portrait—a new career and life story that is significant and meaningful. Contributions to the greater good were encouraged from the perspective of a relational-moral self (with occupation as the central point). In this way it was attempted to construct a relational-moral self that would be unique to each participant.	The purpose of this activity was to create an opportunity during which the participants could narrate their new career-life stories. Inherent life themes were linked to issues that provided personal as well as social meaning.
5	Activities that the participant could convert into concrete action were specified.	The plan concentrated on actions that would address those issues that had initially compelled the participant to seek counseling. Super et al. (1990) [in Savickas' (2011) model] refer to the consecutive actions involved as crystallization (the match between self and a possible profession), specification (a more advanced examination of specific bits of information) and actualization (the choice is actually made by the participant trying it out).	The purpose was to mobilize participants to take steps to sort out their reason for reporting for counseling within the framework of the more holistic picture (life portrait) that had been negotiated by the participant and counselor.
6	Follow-up.	Short-term and long-term follow-up actions were planned, based on (if required) the revision of the exploration outcomes and provisional future decisions.	The purpose was to monitor the progress made by participants and, if necessary, to render further support in respect of Item 5 above.
1–6	Journal entries.	The participants were requested to reflect on aspects of their experiences with regard to the intervention programme and to jot down these thoughts (i.e., an upsurge of emotions while they were completing any of the tasks; as well as thoughts and images that arose about themselves and their lives).	The aim of the reflections during journal entries was to give the participants more profound insights into their career-life stories and contexts. In this way they could identify their life themes and thus eventually sculpture a more coherent and meaningful image of themselves and their career lives.

### Data analysis

The data analysis strategy (data reduction and interpretation) consisted of the following seven phases: organizing the data; absorbing the data; generating deductive categories and themes; coding the data; offering interpretations by means of analytical notes; searching for alternative insights, and presenting the research in the form of a report (Marshall and Rossman, 2011). The themes and subthemes implemented in the research were determined deductively (a priori). Examples of such themes are the concepts “own autonomy” and “commonality,” which were embedded in the construct of the relational-moral identity. However, note was taken of the view held by Bernard and Ryan (2010, p. 107), namely that “no matter how hard we try, there are no purely inductive (or deductive) studies.” Our style of data analysis can therefore best be described as deductive-inductive. Unsurprisingly, a number of additional subsubthemes emerged inductively during the analysis of the data.

Subsubthemes that emerged inductively and were engaged with in an integrated manner, demonstrated that a balanced oscillation between self-centeredness and “other”-centeredness could be negotiated. In addition, it became clear that deliberate diversification (which comprises the combination of virtues and meaningful activities—including a profession) could be fundamental to spiritual or relational-moral identity formation. Crucially, the oscillation between self-centeredness and self-transcendence and the application of core *sparks* (deep-seated passions or interests (Benson, 2008; Benson and Scales, 2009) emerged inductively as fundamental to identity work with the emerging adults in our study. Scales et al. (2010) believe that *sparks* are people's idiosyncratic traits that give evidence of positive values and can be implemented to ignite fires in them to promote prosocial and generative values. In reality, *sparks* indicate “a passion for a self-identified interest, skill, or capacity that metaphorically lights a fire in an adolescent's life, pro-viding energy, joy, purpose, and direction. Thriving is … the combination over time of sparks, and the action that the youth and others take to support, develop, and nurture those sparks” (Scales et al., 2010, p. 264).

### Ethical issues

Permission to conduct the study was obtained from the Ethics Committee of the University of Pretoria's Faculty of Education. We implemented standard measures to ensure participants' wellbeing and to protect them from harm throughout the research. Written informed consent was obtained from participants, and privacy, confidentiality, and anonymity were maintained throughout. Feedback was given to participants at all stages and during all the phases of the research. Moreover, we released our research findings in an acceptable and responsible manner (Piper and Simons, 2005). Since some participants could perhaps manifest behaviors and emotions that would warrant counseling during the course of the research, arrangements were made for counseling to be provided by a fellow educational psychologist who agreed to offer this service should it be required. This was done to ensure the integrity of our role as researchers in this study and to avoid any role confusion.

## Results

### Themes and subthemes

Themes and subthemes (which emerged from the literature study and were used deductively during the research) as well as subsubthemes (which emerged inductively during data analysis) appear in Tables 3, 4 below.

**Table 3:** **Theme 1 and its subthemes and subsubthemes**.

**THEME 1: OWN AUTONOMY (Identity formation of the autonomous “self”)**
Subtheme 1.1: Ideology or world view (background to the life story)
Subsubtheme 1.1.1: A self-fulfilling world view
Subsubtheme 1.1.2: A self-transcending world view
Subtheme 1.2: Key scenes (highlights, lowest points, and turning points)
Subsubtheme 1.2.1: A self-assertive plasticity
Subsubtheme 1.2.2: A self-transcending plasticity (example of a citizen leader included)
Subtheme 1.3: Key images (that promote the vitality of the story line)
Subsubtheme 1.3.1: Intrapersonal diversity
Subsubsubtheme 1.3.1.1: Prosocial objectives
Subsubsubtheme 1.3.1.2: Generative values

**Table 4:** **Theme 2 and its subthemes and subsubthemes**.

**THEME 2: COMMONALITY (Relational-moral identity formation)**
Subtheme 2.1: Family (relatives), friends, and intimacy
Subsubtheme 2.1.1: Recentring of the emerging adult
Subsubtheme 2.1.2: Family as the primary “context” for spiritual development
Subsubtheme 2.1.3: Organized youth groups as additional context for spiritual development
Subsubtheme 2.1.4: The tendency to make choices (in respect of life roles) in terms of where the force of gravity is
Subtheme 2.2: Work or study role
Subsubtheme 2.2.1: Spiritual goals (deeper meaning of the career) that are supplementary to defined measures of success
Subtheme 2.3: Civilian self
Subsubtheme 2.3.1: Community-oriented aspects
Subsubtheme 2.3.2: Political aspects
Subtheme 2.4: Cosmopolitan self
Subsubtheme 2.4.1: Socio-political literacy
Subsubtheme 2.4.2: The importance of systemic reflection on a global level

Life design counseling enabled the researchers to confirm the existence of participants' autonomous self (also referred to as the “own actual self”). This “self” functions in a (daily) relational matrix (complex and mutual; also known as the “commonality aspect” or common/relational self). Aspects of the autonomous self were further differentiated (by means of the deductive confirmation of aspects obtained from the data analysis) into the participants' world views (ideologies); the highlights, lowest points and turning points in their life stories; their own unique characteristics; and their plans, linked to dreams and goals. These differentiated aspects served as subthemes. Subsubthemes that emerged inductively were integrated with the deductively derived themes and subthemes. All the issues were therefore condensed into a framework that enabled the researchers to compare the data in greater detail with contemporary research conducted into the participants' “self-formation,” as well as their growth and development.

In the next paragraphs the findings are presented within the framework (and as example) of the autonomous self on the one hand—and its associated differentiation—in a (daily) relational matrix (complex and mutual) of family and friends; intimacy; work or study role; and the civil and cosmopolitan self (the common self) on the other hand. The findings are subsequently related to the aim of the research in the form of a discussion.

### Own autonomy (the autonomous or own actual self)

#### Ideology or world view (as background to the life story)

The ideologies or world views of the participants were self-fulfilling (self-centered) and/or self-transcending. The following is an example of a self-centered world view taken from the study: *What I want to do with my life is … I want to live my life doing what I want to do. I want to travel. I want to enjoy my life as a human being. I want to live a fulfilled life*. In contrast—an example of a self-transcending world view is the following: *The day we realize that we are living not for ourselves but for the people and the generations to come, is the day we are all free*.

#### Key scenes (highlights, lowest points, and turning points in the life story)

Participants were able to apply self-regulation, which proved in some cases to be self-assertive (self-fulfilling or self- centered) and self-transcending in other cases. The following is an example (translated from Afrikaans) of self-fulfilling orientation: *I think that bankruptcy had a very serious effect on my life. (Ek dink daardie bankrotskap het 'n baie groot effek op my lewe gehad.) It also caused my business thinking to change drastically. (Dit het ook my besigheidsdenke baie verander.) Such as how to tackle things … how to avoid putting everything in one basket … things may go wrong … and to start asking the right questions. (Soos hoe om goeters aan te pak …hoe om alles nie in een plekkie te sit nie …goed kan verkeerd gaan …en die regte vrae te begin vra.)* An example of self-transcending orientation is the following: “*I don't really value money too much, neither do I value materialistic things as prerequisite for my success. But I do value service to other …I would rather achieve my goals at the service for people, not at the expense of others.”*

#### Own unique characteristics and plans, linked to dreams and goals in life

Participants displayed idiosyncratic characteristics that imply positive values (also known as *sparks*) that some of them applied randomly in respect of contributions to society. By deliberately focusing the participants' attention on the presence of their *sparks* and indicating the latter's potential with regard to constructive contributions to society, the concept of deliberate/purposeful diversification was raised. An example of purposeful diversification is the following remark that one of the researchers made toward a participant who shared her personal vision regarding her medical career with this researcher. *So for me, the ‘ideal self’ that I see in you, is the following: That you are more than able to make a supreme and* (researcher repeated the participant's own words) *actually in your own words: ‘making a supreme and startling, and prominent contribution to the development and improvement of mankind’*.

Prosocial and generative actions never take place in isolation—always in context. The latter was categorized in the current research as family, friends and intimacy, work or study role, and the civil or cosmopolitan self. A brief description and examples of the prevalence of prosocial and generative values in the different contexts follow next.

### Commonality (the common or relational self)

#### Family and friends

Family appeared to constitute the primary context for spiritual development. Furthermore, it seemed that an organized youth group (consisting of friends, mentors and youth leaders) could well provide an additional context for spiritual development. An example of the parental home as primary context for spiritual development is the following: *It will also be my father. Because everything that he did when he was young is now a light to my path*. The following is an example of an organized youth group that serves as an additional context for spiritual development: *We are a few young people that have a home cell together every week on Wednesdays and Fridays where we discuss spiritual topics and encourage one another*.

#### Intimacy

Intimacy among emerging adults was found to be fragile/delicate and fragmented. An example of the above is the following remark by a participant (translated from Afrikaans): *The girl with whom I was …we were together on and off for seven years and then in April … everything eventually came to an end. (Die meisie saam met wie ek was …ons was aan-en-af vir sewe jaar en ons het toe in April …toe nou uiteindelik tot 'n einde gekom.) I am sad about it, because it is like, I was very down because it had been seven years of my life, and it is someone whom I really love and still love her, but it just didn't work. (Dis vir my sad, want dis soos, ek was baie down gewees want dit was sewe jaar van my lewe, en dis iemand vir wie ek regtig lief is en ek is nog steeds lief vir haar, maar it just didn't work)*.

#### Work or study role

A spiritual approach or orientation seemed to be beneficial for employees, especially in view of the fact that emerging adults are experiencing challenges to stabilize themselves economically. The following comment by a participant is an example of a spiritual approach or orientation: *I also like working with customers and sometimes I would go the extra mile helping them and there are customers who always ask for me when they want to buy something. There is also some staff that has been in the company for years even before me. But the manager always calls me instead for information*.

#### Civilian self

It became evident that participants displayed a generous degree of community-oriented attitudes and objectives. The following is an example: *Like in my culture we used to say, ‘umutu abantu omuntu’ …it means we must learn how to share with other people. That way we can help one another to go far*. According to participants, community-oriented activities could be far more effective if the government were to become involved in a meaningful manner: *Everyone must be involved in these issues of contributing and the government should facilitate the project*. The participants also indicated that they did not trust politics as it was being practiced at present: *I don't have any interest in politics. They don't have a mission or vision. They are just stagnant. They are in the parliament to benefit themselves, not for the interest of the country*.

#### Cosmopolitan self

Findings with regard to the effect of globalization on emerging adults showed that participants were aware of the interconnectedness of the world: *Yes, we are interconnected. With technology, you can be anywhere and anytime. [And] for example, if one country is going through challenges, we will also experience the same thing because we are dealing with one another*. The participants also indicated that they possessed a critical social awareness: *If you say to them, ‘Angola, panga and diamonds’, then they also look at you in a strange way. (As jy vir hulle sê, ‘Angola, panga en diamante,’ dan kyk hulle jou ook snaaks aan.) Then they also don't know what you are talking about …only ‘they do this and this and this’. (Dan weet hulle ook nie waarvan jy praat nie …net ‘hulle doen dit en dit en dit.’) They do not realize that they don't even have a notion of what happened earlier. (Hulle besef nie hulle het nie eers 'n idee van wat vooraf gebeur het nie.)* Emerging adults furthermore seem to be aware of the value of greater collaboration among and involvement of individuals on a cosmopolitan level: …*but the same thing happened in Egypt and Syria…. 30 years ago it would have been impossible for those rebellions to happen, because those people did not know it was possible. (…maar dieselfde ding het gebeur in Egipte en Sirië…daai opstande sou nie 30 jaar terug kon gebeur het nie, want daai mense het nie geweet dit is moontlik nie.) ‘We thought we were living in Egypt and we are being oppressed’ and that's it …but now they have seen on the news and on Facebook and such places that it is possible to stand up. (‘Ons het gedink ons bly in Egipte en ons is onderdruk’ en dit is so …maar hulle het nou op die nuus en op Facebook en op sulke plekke gesien dit is moontlik om op te staan)*.

## Discussion

Our findings suggest that life design counseling enhances emerging adults' self-awareness and relational-moral self. The finding with regard to an “autonomous-relational” participant corresponded with the findings of other researchers (Markus and Kitayama, 1991; Bakan, 1966; Deci and Ryan, 2000, 2008; Kagitcibasi, 2012; Inguglia et al., 2014). All these researchers found that autonomous behavior (which involves mainly self-management) and relational behavior (commitment to others in a commonality sense) are fundamental needs experienced by all people. These needs are considered as highly complementary motives and the integration of the two are regarded as essential for optimal psychological well-being. The notion of the “autonomous (own actual) self” is extended to autonomous “self”-identity formation, while the “relational self” is extended to relational-moral identity formation (McAdams, 1985; Gadow, 1999; Parker, 2007; Barrett, 2013; Roberts and Côtê, 2014). The concepts “own autonomy” and “commonality,” coupled with “autonomous self” identity formation and “relational-moral identity formation” served as the two main themes in the current research.

McAdams and Cox (2010) further differentiated own autonomy as a “self' operating in three aspects, namely: the self as *author* (of the own narrative identity) who is able to ponder and reflect on the self as *actor* (with his/her unique properties as obtained from the key images of the participants” life stories) and the self as *agent* (the autonomous self with future plans and goals). The autonomous self (as agent) operates from a specific ideology or world view and with a particular resilience (as obtained from the key images of the participants' life stories) in a daily (relational) matrix. In summary, the participants' ideologies or world views, together with the key images and key scenes from their life stories, served as subthemes.

As stated previously, a number of subsubthemes emerged inductively. It became apparent that the ideologies or world views of participants could be negotiated as a balanced oscillation between “self-centredness” and “other-centredness,” and that that purposeful diversification or the combination of sparks (Benson, 2008) (especially idiosyncratic traits that confirm the presence of positive values) and meaningful activities (including the pivotal actions of choosing and executing a career) may be fundamental to spiritual identity formation. In terms of an occupation, it seemed that when participants consider work a calling, this may boost the dynamic interaction (mutually beneficial and adjustable relationship) between employee and the workplace—in other words, both employee and context may benefit from the interaction. This finding supports the findings of Adams (2012), Colozzi and Colozzi (2000), Dik and Duffy (2009), and Dik et al. (2009), who confirm the powerful influence of the belief that one has a “calling” for a career or that one's career is meaningful or useful or beneficial to others.

The family proved to be the primary context for spiritual development. Shulman and Connolly (2013) argue in this regard that the increased investment of energy by emerging adults in work or study may perhaps exert pressure on their relationships, which culminate in the postponement of marriage, instability in relationships and the hesitation to conclude agreements. These aspects seem to be characteristic of the moratorium phase of emerging adults, during which romantic commitments and life plans—more specifically, the different facets of the lives of emerging adults—are co-ordinated. Their choices of life roles therefore incline toward the place where gravity lies during this phase (Shulman and Connolly, 2013).

Although it seemed that the participants had a generous degree of community-orientated attitudes and goals, “political” involvement was labeled as “atomized” activity. Thus, the possibility that the social capital of emerging adults could be wasted is becoming an increasing and alarming reality. In this regard it seems that organized youth groups could perhaps provide a temporary context within which the need and potential of emerging adults to become involved in community projects could be stimulated.

All things considered, Flum (2015) contends that being in the world does not only imply relational and contextual connotations [the actor and agent suggested by McAdams and Cox (2010)]; it also involves a retrospective and prospective view of the self (McAdams and Cox's, 2010 author). Lombardo (2007) refers to a positive and informed narrative vision or picture that can be converted into action—an issue that was discussed as “life design counseling.”

In brief, it seems that the self cannot be independent of the own social-historical existence (the relational and contextual aspects of the own life story). By means of a retrospective as well as a prospective view of the self, a map of possible roles and potential roles may be provided within which new thoughts, actions and self-definition are desired, allowed, feasible, wanted, and essential (Flum, 2015). By making the intertwined mutual relationship among the self, the proximal and distal community more explicit, the individual is more inclined to exercise own autonomy in favor of commonality (a relational-autonomous relationship), to which the spiritual dimension of prosocial and generative actions may be added (a relational-moral autonomous self).

We found no evidence that the deliberate promotion of a balanced oscillation between “own autonomy” and “commonality” by way of life design counseling has been reported before. More specifically, no evidence was found of the mutualistic design of a life career within the larger framework of the purposeful promotion of own autonomy (the stimulation of a balance between self-fulfillment and self-transcendence through the purposeful and intelligent application of *sparks*, both for own wellbeing and for the wellbeing of the community) and commonality. These findings underscore the importance of using career guidance- and education-related dialogues to promote oscillation between emerging adults' “own autonomy” and “commonality”and to advance their career-life-design within the larger framework of the purposeful promotion of own autonomy and commonality in service of fair and sustainable human development globally (Guichard, 2013). Given the increasing feeling of isolation among many workers, and emerging adult workers in particular, as well as the belief and experience that their needs are not being met to a satisfactory extent in work contexts, we believe that research on this topic is vitally important.

### Limitation of the study

The somewhat bounded social class of the sample represents a limitation.

### Recommendations for future research

The findings of our study may have important implications for career counseling in other contexts. Firstly, it is important to conduct research on possible ways in which this type of counseling can be administered in group-based contexts. Second, longitudinal research to trace the medium- and longer-term effect of life design counseling on participants is essential. Third, it is essential to ensure that this type of intervention is made available to the millions of people who are currently denied virtually any kind of career counseling related intervention. Lastly, researchers should be wiling to alter and adapt the intervention described here to see which intervention works best in different contexts and to report on their findings.

## Conclusion

Our findings further the view expressed by Taylor (1989, p. 197), who remarked that “in order to make minimal sense of our lives, in order to have an identity, we need an orientation to the good, and we see that this sense of the good has to be woven into my understanding of my life as an unfolding story.” Moreover, our findings support Lombardo's (2007) timeless observations that wise individuals regard their own identity formation as an ongoing construction, that the core of their being involves a creative force and that they see the future as an opportunity for further growth, but also as an opportunity to advance the growth of “others.” Promoting these sentiments is particularly central in the context of the construction of a relational-moral self, and associatively, in the construction of a career-life identity. Dik et al. (2012) rightly argue that career counseling should strive to involve individuals in meaningful work that offers them opportunities to promote social harmony directly and indirectly.

The findings pertaining to community-oriented aspects in this article further the observation of Arnett (2013, p. 5), who argues that “today's emerging adults are not Generation Me but Generation We, an exceptionally generous generation that holds great promise for improving the world.” These findings are accurately represented by the concepts *iSintu* (beholding the “self-identity” in the “other”) (Nussbaum et al., 2010), as well as the term *ubuntu*, which suggests the human commitment and interdependence experienced in neighborhoods, organizations and the global community (Le Grange, 2012).

In a nutshell—this article suggests that the value of life design counseling can be summarized by combining the remarks made by Buechner (1993, p. 119) and Savickas (2006, 2011, p. 13), namely: *The magic in one's career life happens where your deep gladness and the world's deep hunger meet*. Harnessing life-design counseling (for career construction) to help all people (emerging adults in particular) challenged by unemployment and poverty become employable, find decent work and enhance their sense of self offers society its best chance to achieve this laudable aim, and, in doing so, to promote the idea of a fair and just society (Maree and Di Fabio, 2015).

## Author contributions

JM and AT conducted the research, while JM wrote the manuscript and received substantial input from AT.

### Conflict of interest statement

The authors declare that the research was conducted in the absence of any commercial or financial relationships that could be construed as a potential conflict of interest.
